# Direct conversion of human fibroblasts into retinal pigment epithelium-like cells by defined factors

**DOI:** 10.1007/s13238-013-0011-2

**Published:** 2014-01-29

**Authors:** Kejing Zhang, Guang-Hui Liu, Fei Yi, Nuria Montserrat, Tomoaki Hishida, Concepcion Rodriguez Esteban, Juan Carlos Izpisua Belmonte

**Affiliations:** 1 Gene Expression Laboratory, Salk Institute for Biological Studies, 10010 North Torrey Pines Road, La Jolla, CA 92037 USA; 2 National Laboratory of Biomacromolecules, Institute of Biophysics, Chinese Academy of Sciences, Beijing, 100101 China; 3 Department of Molecular and Cellular Physiology, Stanford University School of Medicine, 265 Campus Drive, Stanford, CA 94305 USA; 4 Center for Regenerative Medicine in Barcelona, Dr. Aiguader 88, 08003 Barcelona, Spain; 5 Biomedical Research Networking Center in Bioengineering, Biomaterials and Nanomedicine (CIBER-BBN), Barcelona, Spain

**Keywords:** retinal pigment epithelium, fibroblasts, direct conversion

## Abstract

The generation of functional retinal pigment epithelium (RPE) is of great therapeutic interest to the field of regenerative medicine and may provide possible cures for retinal degenerative diseases, including age-related macular degeneration (AMD). Although RPE cells can be produced from either embryonic stem cells or induced pluripotent stem cells, direct cell reprogramming driven by lineage-determining transcription factors provides an immediate route to their generation. By monitoring a human RPE specific Best1::GFP reporter, we report the conversion of human fibroblasts into RPE lineage using defined sets of transcription factors. We found that Best1::GFP positive cells formed colonies and exhibited morphological and molecular features of early stage RPE cells. Moreover, they were able to obtain pigmentation upon activation of Retinoic acid (RA) and Sonic Hedgehog (SHH) signaling pathways. Our study not only established an ideal platform to investigate the transcriptional network regulating the RPE cell fate determination, but also provided an alternative strategy to generate functional RPE cells that complement the use of pluripotent stem cells for disease modeling, drug screening, and cell therapy of retinal degeneration.

**Electronic supplementary material:**

The online version of this article (doi:10.1007/s13238-013-0011-2) contains supplementary material, which is available to authorized users.

## Introduction

The retinal pigment epithelium (RPE) is a pigmented monolayer of epithelium residing outside of the neurosensory retina where it supports metabolic and cellular processes of retinal photoreceptors. Dysfunction and degeneration of RPE lead to photoreceptor loss in many sight-threatening diseases, including the leading causes of blindness in the developed world, age-related macular degeneration (AMD) (Khandhadia et al., 2012). Currently, treatments available for these diseases are limited and do not offer a cure for the cell loss. Therefore, the generation of functional RPE cells is of great therapeutic interest to the field of regenerative medicine and offers possible cures for retina degeneration diseases (Schwartz et al., 2012).

Induced RPE differentiation has been achieved previously using embryonic stem cells (ESCs) and induced pluripotent stem cells (iPSCs) (Carr et al., 2009; Lu et al., 2009; Zhu et al., 2013). Although differentiating RPE cells from hESCs is promising (Zhang et al., 2013), the application of hESCs for therapeutic purposes remains challenging due to un-eased ethical concerns. iPSC technology holds the potential to be used to generate patient specific cells for autologous cell transplantation, which has created enormous expectations and circumvented some ethical debates (Takahashi and Yamanaka, 2006; Takahashi et al., 2007). However, differentiation of RPE cells from iPSCs is inefficient, time-consuming, and variable among different iPSC lines (Buchholz et al., 2009). Moreover, iPSCs present several safety concerns such as the genetic and epigenetic aberrations they are carrying, as well as the potential risk for tumor formation (Panopoulos et al., 2011). Recently, advances in direct cell lineage conversion have suggested a potential solution to these issues. The fact that one somatic cell type can be readily converted into another implicates access to abundant resources of any clinically relevant cell type. Moreover, as direct lineage conversion bypasses the pluripotent state, it could theoretically reduce the risk of tumorigenicity after transplantation (Ben-David and Benvenisty, 2011). Using distinct sets of transcription factors, direct lineage conversion technology has been applied to produce various cell types, including neurons, hepatocytes and neural stem cells (Vierbuchen et al., 2010; Huang et al., 2011; Kim et al., 2011; Pang et al., 2011; Sekiya and Suzuki, 2011; Giorgetti et al., 2012; Liu et al., 2012; Zhang et al., 2012).

Here we report the successful development of a RPE-specific Best1::GFP reporter, which faithfully represented human RPE lineage commitment during hESC differentiation. Using this reporter system, we show that a defined set of transcription factors can reprogram human fibroblasts into Best1::GFP^+^ colonies. These Best1::GFP^+^ cells exhibit specific morphological and molecular features of RPE lineage and are capable of pigmentation. Our study not only provided a powerful system to study the nature of cellular identity and plasticity of RPE lineage, but also offered a new path to produce functional RPE cells for regenerative therapy and drug development in the future.

## Results

### Establishment of a human RPE specific reporter system

**Figure 1:**
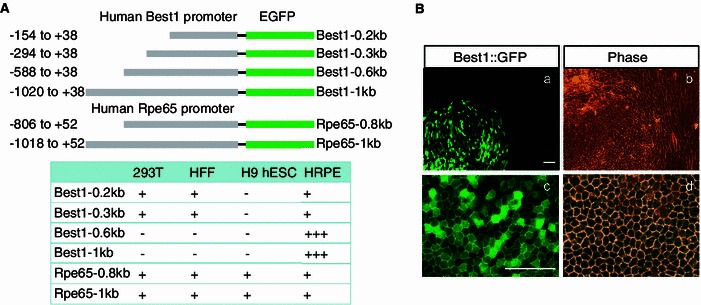
Generating human RPE specific reporters. (A) Schematic representation of the promoter regions of human *Best1* or *Rpe65* gene for reporter constructs. The table shows the relative GFP levels of each reporter observed in different cell lines. HRPE, human primary RPE cells; -, no expression; +, weak expression; +++, strong expression. (B) Best1::GFP specifically expressed in RPE-like cells differentiated from H9 hESC. All scale bars: 100 μm

A proper reporter system is essential for monitoring efficient cell fate conversion in reprogramming studies. A series of truncated versions of human *Bestrophin1* (*Best1*) gene and *Rpe65* gene promoter, have been shown to mediate robust ocular reporter gene expression that is restricted to the RPE in transgenic mice (Boulanger et al., 2000; Esumi et al., 2004; Acland et al., 2005; Esumi et al., 2009). To test their functionality in human cells, these promoter fragments were cloned into the upstream of EGFP in a lentiviral vector, pGreenZeo (Fig. 1A). We then performed lentivirus infection to test the promoter specificity in different human cell types including human foreskin fibroblasts (HFF), HEK293T cells, H9 hESCs, as well as human primary RPE cells (HRPE). Distinct from the non-specific expression of the Rpe65(0.8k) or Rpe65(1k) reporter in all of the examined cell types, the Best1(0.6 kb) (spanning from -588 to +58) and Best1(1 kb) (spanning from -1020 to +38) reporters exhibited high selectivity in driving strong EGFP expression in human RPE cells (Fig. 1A). In the following studies, data presented were generated using Best1(0.6 kb) reporter as Best1::GFP reporter, if not specifically indicated. In order to further confirm the specificity of the reporter, we tested the reporter in H9 ESC differentiated cells at day 60, using a reported protocol (Meyer et al., 2009), which gave rise to a mixed population of various cell types including RPE cells. We observed that the Best1::GFP reporter was only expressed in RPE-like cells, which displayed a morphologically characteristic hexagonal shape, but not in other neural or fibroblast-like cells (Fig. 1B).

**Figure 2:**
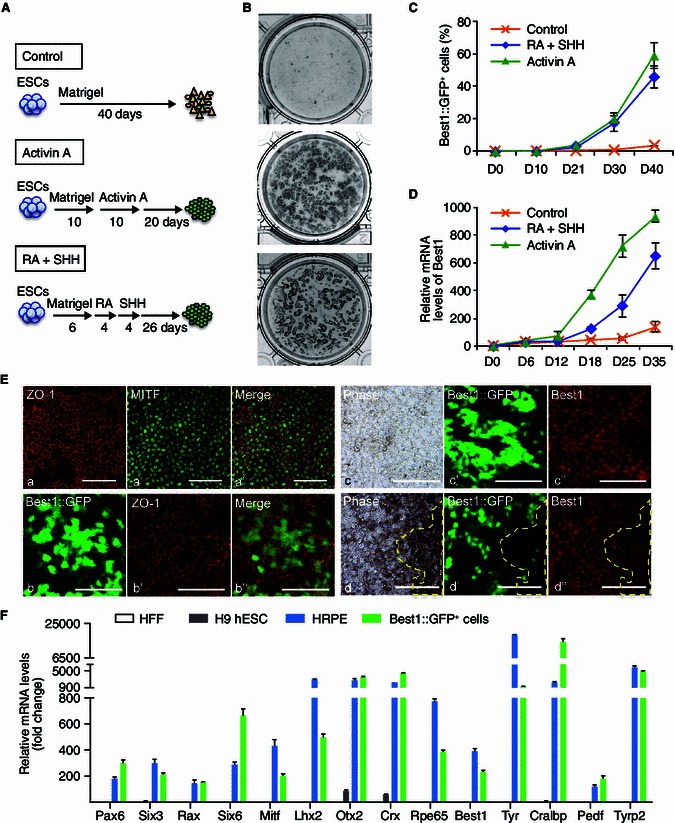
Characterization of Best1::GFP positive cells during hESC differentiation towards RPE. (A) Schematic illustration outlining differentiation of H9 hESCs with Best1::GFP reporter into RPE under different conditions. (B) Pigmentation of RPE cells differentiated from H9 hESCs using three conditions at day 40. (C) Percentages of Best1::GFP^+^ cells calculated by FACS analysis in indicated days of H9 hESC RPE differentiation, with different treatments as indicated in Fig. 2A. Data are presented as mean ± s.d. of three biological replicates. (D) Q-PCR analysis of *Best1* mRNA expression levels in indicated days of RPE differentiation. Expression was normalized to levels in undifferentiated H9 hESC. (E) Immunofluorescence staining and phase contrast microscopy of RPE differentiation from H9 hESCs. a–a’’, Immunofluorescence analysis of ZO-1 and MITF expression at day 45. b–b’’, Best1::GFP and ZO-1 expression at day 45. c–c’’, Best1::GFP expression and immunostaining of Best1 protein. Best1::GFP is highly expressed in non-pigmented or slightly pigmented cells at day 30. d–d’’, Best1::GFP expression and immunostaining of Best1 protein at day 45. Yellow dash line highlighted area indicates diminished Best1::GFP expression in heavily pigmented cells. All scale bars: 100 μm. (F) Relative fold changes of RPE specific genes in HFF, H9 hESCs, HRPE cells and Best1::GFP^+^ cells which were sorted by FACS at day 40 of hESC RPE differentiation. Relative expressions were normalized to levels in HFF

We next evaluated whether the Best1::GFP reporter works properly during the directed RPE differentiation from hESCs. Accordingly, H9 hESCs were transduced with Best1::GFP lentiviral reporter and ten individual colonies were chosen to verify the integration of Best1::GFP expression cassette by genomic DNA PCR (data not shown). Positive colonies were subcultured and expanded for further studies. For RPE differentiation, we developed a rapid and efficient protocol, which is modified from two recent publications (Boucherie et al., 2013; Zhu et al., 2013) (Fig. 2A, group Activin A). With this approach, pigmented cells appeared in a monolayer form 3 weeks after Activin A treatment. Immunostaining showed that these cells expressed typical RPE markers including microphthalmia-associated transcription factor (MITF) and Best1, as well as a tight junction protein, ZO-1, indicating a successful commitment of RPE from hESCs (Fig. 2E). Given the reported roles of Retinoic acid (RA) and Sonic Hedgehog (SHH) signaling in RPE maturation and pigmentation (Okada et al., 2004; Zahabi et al., 2012), we set up an RA plus SHH experimental group. As shown in Fig. 2A and 2B, RA plus SHH treatment enhanced the pigmentation despite resulting in a lower yield of RPE cells compared to the Activin A treatment group.

Monitoring the Best1::GFP expression represents a direct way to visualize the dynamics of RPE differentiation. In both the “Activin A” and “RA plus SHH” treatment groups, Best1::GFP^+^ cells were initially observed at around day 21 of differentiation, and the percentage of Best1::GFP^+^ cells increased to approximate 50% after 40 days of differentiation (Fig. 2C). Consistently, time-course studies showed that the levels of *Best1* mRNA were strongly upregulated after 3 weeks of differentiation from H9 hESCs (Fig. 2D). Interestingly, Best1::GFP was highly expressed in non-pigmented and lightly pigmented RPE cells, while when cells were heavily pigmented, GFP fluorescence diminished or even became undetectable (Fig. 2E c–c″, d–d″). Since endogenous Best1 protein was still expressed in highly pigmented cells (Fig. 2E d–d″), the absence of GFP fluorescence in these cells may be attributed to the absorption of fluorescence by melanin. To further characterize the mature status of Best1::GFP^+^ cells that followed the protocol with Activin A treatment, we performed qPCR analysis at day 40. The results demonstrated that Best1::GFP^+^ cells did express the early eye field genes including *Pax6*, *Lhx2*, *Six6* and *Rax* (Zuber et al., 2003) (Fig. 2F). Two key genes for RPE differentiation, *Mitf* and *Otx2* (Boulanger et al., 2000; Steingrimsson et al., 2004), were markedly upregulated. Other mature RPE hallmark genes were also highly expressed in Best1::GFP^+^ cells, including *Best1*, pigment epithelium derived factor (*Pedf*), RPE-specific protein 65 kDa (*Rpe65*), cellular retinaldehyde-binding protein (*Cralbp*) involved in vitamin A metabolism, tyrosinase (*Tyr*) and tyrosinase related protein 2 (*Tyrp2*), both involved in pigment synthesis (Martinez-Morales et al., 2004; Strauss, 2005) (Fig. 2F). These gene expression patterns of Best1::GFP^+^ cells are similar to those of human primary RPE cells (Fig. 2F). Collectively, these data demonstrate the successful establishment of a human RPE specific reporter system, which faithfully represented RPE lineage commitment during ESC differentiation.

### Definition of transcription factor pool

**Figure 3:**
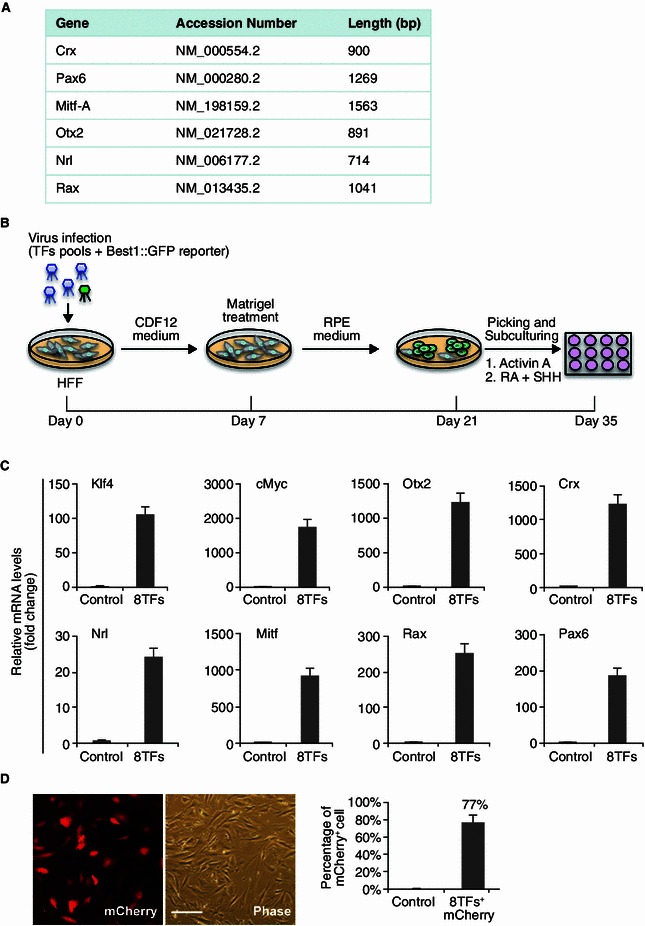
Strategy for transcription factors-mediated human fibroblast to RPE transdifferentiation. (A) Table listing the selected 6 transcription factors (6TFs). (B) Experimental design to induce the conversion of HFF into RPE. (C) qPCR analysis of individual exogenous TF expression 3 days after HFFs were transduced with 8TFs. HFF cells transduced with mCherry were included as the control. Data are presented as mean ± s.d. of three replicates. (D) mCherry as indicator for the transduction efficiency of HFF cells 3 days after retroviral 8TFs plus mCherry were infected. The percentage of mCherry positive cells was indicated. Scale bars: 100 μm

We hypothesized that transcription factors known to instruct RPE formation during development might also facilitate the conversion of other somatic cell types into RPEs. To test this idea, we constructed retroviral expression vectors encoding six candidate transcription factors (TFs): *Rax*, *Crx*, *Pax6*, *Mitf*, *Otx2*, and *Nrl* that have been reported to participate in different stages of retina development and RPE specification (Zuber et al., 2003; Martinez-Morales et al., 2004; Steingrimsson et al., 2004) (Fig. 3A). After transduction of HFF with a combination of these 6TFs together with Best1::EGFP reporter lentivirus, cells were cultured with hESC culture medium (CDF12) to prime the lineage conversion for 7 days. The cells were subsequently cultured in a similar condition that was used to induce RPE differentiation from hESCs (Fig. 3B). However, such a strategy resulted in neither obvious morphological change nor Best1::GFP^+^ cells from parental HFF. This raised a possibility that other factors may be required to increase the epigenetic “plasticity” of the original human fibroblasts. Based on the “clue” that two “Yamanaka factors” (*cMyc* and *Klf4*) are highly expressed in adult RPEs (Salero et al., 2012), we supplemented *cMyc* and *Klf4* into the previous six transcription factor cocktail. qPCR analysis verified the expression of all eight transcription factors (8TFs) in HFF transduced with this new cocktail for three days (Fig. 3C). Using the co-transduced retroviral vector encoding mCherry as an indicator, we found that the retrovirus infection system in HFF achieved a transduction efficiency for individual factors of ~77% (Fig. 3D). This experiment suggests that almost 12% of parental HFF cells could have the opportunity to express all of the eight TFs to initiate the reprogramming process.

### Best1::GFP^+^ cells generated by direct reprogramming of human fibroblasts

**Figure 4:**
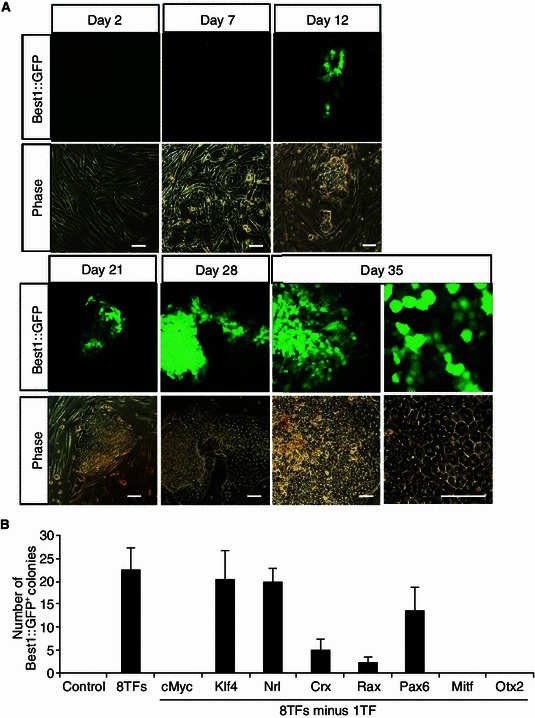
Eight transcription factors induced Best1::GFP^+^ colonies from human fibroblasts. (A) Morphological changes of HFF during the process of RPE lineage conversion. Best1::GFP^+^ cells exhibited cobblestone-like morphology at day 35. All scale bars: 100 μm. (B) Effects of individual factor withdrawal from 8 transcription factors (8TFs) on Best1::GFP^+^ colony formation. HFF cells transduced with mCherry were served as the control. Data are presented as mean ± s.d. of three replicates

We next performed the lineage conversion experiments by transduction of HFF with 8TFs. Encouragingly, a number of Best1::GFP^+^ colonies emerged at 12 days post-transduction. These Best1::GFP^+^ cell colonies kept actively proliferating and became tightly compacted with a high cytoplasmic to nuclear ratio at day 21. Later, Best1::GFP^+^ colonies gradually spread out on matrigel and exhibited a cobblestone-like morphology at day 35 (Fig. 4A). These morphological observations suggested a RPE progenitor-like status of the generated Best1::GFP^+^ cell colonies. To determine which of the eight factors were necessarily required for generating Best1::GFP^+^ colonies, we removed each TF-encoding vector from the 8TFs cocktail one at a time (Fig. 4B). As shown in Fig. 4B, excluding *cMyc*, *MitfA* or *Otx2* eliminated GFP^+^ colony formation, and the number of Best1::GFP^+^ colonies was dramatically reduced when *Rax*, or *Crx* was removed. Interestingly, when *Pax6* was removed, the number of Best1::GFP^+^ colony slightly reduced, while the number of colonies was not discernibly changed when *Klf4* or *Nrl* was ruled out (Fig. 4B). However, when any combination of two or three factors of *Klf4*, *Pax6* and *Nrl* was removed from the 8TFs pool, we could not detect any Best1::GFP^+^ colonies (data not shown). These data indicated that *cMyc*, *Mitf*, *Otx2*, *Rax*, and *Crx* are crucial for reprogramming of human fibroblasts into Best1::GFP^+^ cells, while *Klf4*, *Nrl* and *Pax6* individually are not so important and could be omitted or replaced by other factors.

### Characterization of HFF-derived Best1::GFP^+^ cells

**Figure 5:**
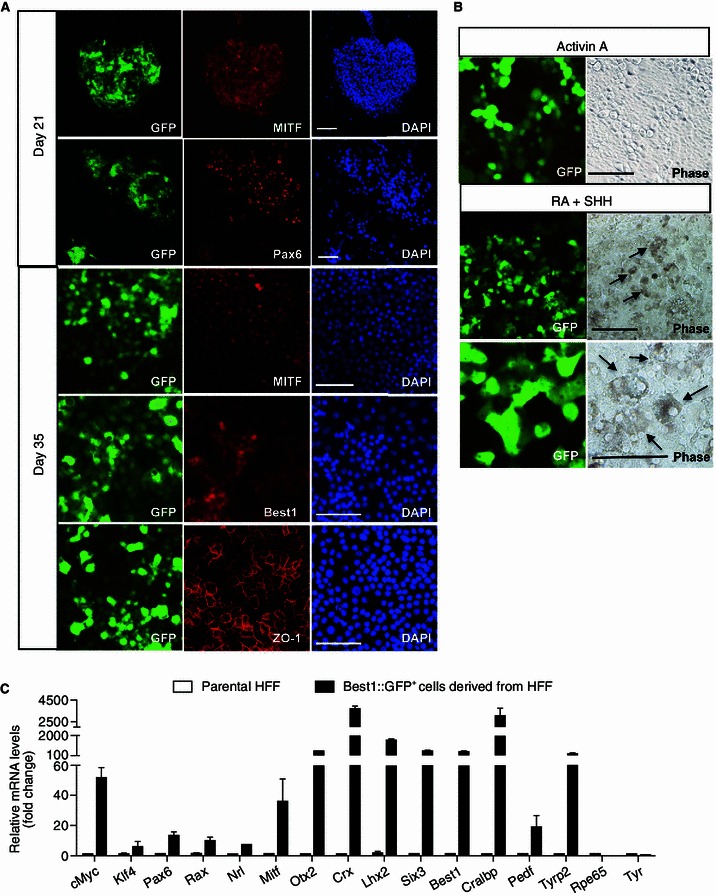
Characterization of Best1::GFP^+^ cells converted from human fibroblasts. (A) Immunostaining of MITF, Pax6, Best1 and ZO-1 in Best1::GFP^+^ colonies. DAPI was used to counterstain the nuclei (blue). All scale bars: 100 μm. (B) Pigmented cells indicated by arrows were observed at day 35 post-transduction in the Best1::GFP^+^ colonies generated by treatment with RA plus SHH, but not by Activin A treatment. All scale bars: 100 μm. (C) qPCR analysis of RPE-specific gene expression. 8TF-induced Best1::GFP^+^ colonies from HFF were picked and subcultured in RA plus SHH condition. Best1::GFP^+^ cells were sorted for qPCR analysis at day 35. Relative expressions were normalized to levels in parental HFF. Data are presented as mean ± s.d. of three replicates

To assess whether Best1::GFP^+^ colonies hold the potential to achieve known characteristics of cultured RPE cells, we evaluated the molecular signatures of the generated Best1::GFP^+^ cells induced by 8TFs. Immunofluorescence analysis demonstrated that most of the Best1::GFP^+^ colonies were positive for MITF staining (Fig. 5A). We observed that only some of the colonies expressed Pax6, which is consistent with the notion that down-regulated expression of *Pax6* occurs upon RPE maturation (Fig. 5A and data not shown). To induce the further maturation of the Best1::GFP^+^ colonies, we picked the Best1::GFP^+^ colonies onto matrigel coated wells at day 21, and then the cells were maintained in the medium consisting of Activin A alone, or RA plus SHH (Fig. 3B). Both conditions promoted further growth of the Best1::GFP^+^ colonies. At day 35 post-transduction, cells derived from Best1::GFP^+^ colonies exhibited cobblestone-like morphology resembling RPE cells differentiated from hESCs, and expressed RPE-specific markers including MITF, ZO-1 and Best1 (Fig. 5A). More importantly, in the RA plus SHH treatment group, pigmented cells were observed at day 35, indicating that Best1::GFP^+^ cells have achieved further maturation giving rise to functional RPE cells (Fig. 5B). To further characterize their identity, we purified the Best1::GFP^+^ cells by flow cytometry analysis (FACS), and performed qPCR to analyze the transcripts of RPE-specific factors. Compared to the control HFF cells, the generated Best1::GFP^+^ cells expressed not only the transcription factors we introduced, but also other eye field genes such as *Six3* and *Lhx2*. Additionally, a series of mature RPE marker genes, including *Best1*, *Cralbp*, *Pedf*, and *Tyrp2*, were also dramatically induced in Best1::GFP^+^ cells directly converted from HFF, and notably these genes are involved in different functions of RPE, e.g. vitamin A metabolism, pigment synthesis and growth factor secretion etc. (Strauss, 2005) (Fig. 5C). Nevertheless, it should be noted that some of the mature RPE markers such as *Rpe65* and *Tyr* still remained at low expression levels, suggesting the functionally incomplete maturation of these Best1::GFP^+^ cells at this stage (Fig. 5C). Taken together, these results indicate that Best1::GFP^+^ colonies generated from human fibroblasts recapitulated the morphological and molecular features of RPE cells, which, upon further maturation, may represent an invaluable RPE replacement for the field of regenerative medicine.

## Discussion

Encouraged by the first report of the positive implication from prospective clinical trials transplanting hESCs derived RPE cells into two patients (Schwartz et al., 2012), RPE cells generated from pluripotent stem cells have attracted increasing attention for the promise of regenerative medicine (Zhang et al., 2013). Current methods differentiating RPE cells from human pluripotent cells include spontaneous differentiation, monolayer differentiation or 3D culture using mouse and human ESCs (Meyer et al., 2009; Osakada et al., 2009; Nakano et al., 2012). Although several of these recent methods have significantly advanced the yield and accelerated differentiation, all methods to date result in a mixture of RPE cells and neural retina cells, and thus require stringent selection before any therapeutic application. However, the only method of RPE selection described so far is the manual picking and expansion of pigmented cells, a time and labor consuming process. In this study, we generated a RPE specific reporter, which faithfully represents RPE cell differentiation and allows for easy cell sorting.

Lineage conversion of one somatic cell type to another is an alternative approach for generating specific cell types bypassing a pluripotent state. This approach has been successful in various cases, from neural lineages to cardiomyocytes and hepatocytes (Sancho-Martinez et al., 2012; Yi et al., 2012). Here we show that a small set of transcription factors can convert adult human fibroblasts into Best1::GFP positive cells, which bear many morphological and molecular features of RPE such as the expression of *Mitf*, *Best1*, *Cralbp*, and *Tyrp2*. Moreover, these cells are able to obtain pigmentation, a typical characteristic of functional RPE cells.

Although we cannot rule out the possibility that other RPE-inducing factors have been overlooked, our study indeed established a platform to study the transcriptional network that regulates the conversion from human fibroblasts to RPE cells. Consistent with the previous reports that Mitf and Otx2 are crucial for RPE development and function (Martinez-Morales et al., 2004; Bharti et al., 2006), our data showed that Mitf and Otx2 are necessary for Best1::GFP^+^ colony formation. Notably, mature RPE cells are polarized, highly pigmented cells which have very limited ability for proliferation, a feature which is not suitable to be used for cell transplantation (Salero et al., 2012). On the other hand, Best1::GFP^+^ colonies we converted from adult fibroblasts showed a progenitor-like status which is expandable. Developing an inducible expression system capable of shutting down some progenitor genes’ expression at certain time points would be crucial for controlling the switch between the proliferation and maturation status of these Best1::GFP^+^ cells. In addition to transcription factors, culture condition has also been highlighted as a critical factor determining the lineage conversion process (Efe et al., 2011; Kurian et al., 2013). Here in this report, we provided a matrigel based culture condition combined with RA and SHH treatment that supports the generation of pigmented cells from the Best1::GFP^+^ cells. Although we have not tested whether the RPE cells we obtained are fully functional, e.g. by cell transplantation in animal models, with further methodological optimization, this approach would facilitate the functional RPE cells’ generation from human fibroblasts. In summary, our findings provide a powerful system not only for studying the molecular nature of cell identity and plasticity, but also for developing therapeutic strategies for retinal degenerative diseases.

## Material and methods

### Cell culture

H9 and H1 hESCs (WiCell Research) were maintained on a layer of mitotically inactivated mouse embryonic fibroblasts (MEFs) in hESC medium (CDF12 medium): DMEM/F12 (Invitrogen) supplemented with 0.1 mmol/L non-essential amino acids (Invitrogen), 1 mmol/L GlutaMAX (Invitrogen), 20% Knockout Serum Replacement (Invitrogen), 55 μmol/L β-mercaptoethanol (Invitrogen) and 10 ng/mL bFGF (Joint Protein Central). hESCs were also cultured in mTeSR medium (StemCell Technologies). Plates were coated with Matrigel (BD Biosciences), and medium was changed daily.

Human primary RPE cells (HRPE) (Lonza) used as a positive control were cultured in basic RPE medium consisting of DMEM/F-12 supplemented with 0.1 mmol/L non-essential amino acids, 1 mmol/L GlutaMAX, 1% N2 supplement (Invitrogen) and 5% Knockout Serum Replacement (Invitrogen).

### RPE differentiation from hESCs

hESCs were dissociated as cell clumps and plated on 1% ESC qualified-Matrigel for 1 h. Attached aggregates of cells were covered with 2% ESC qualified-Matrigel diluted in N2B27 medium consisting of DMEM/F-12 supplemented with 0.1 mmol/L non-essential amino acids, 1 mmol/L GlutaMAX, 1% N2 supplement (Invitrogen) and 2% B27 (Invitrogen). After overnight incubation, fresh medium without Matrigel was added and changed every other day.

For RA plus SHH treatment, Retinoic acid (RA) (500 nmol/L, Sigma) was supplemented into N2B27 medium from day 7, then gradually reduced to 200 nmol/L by day 10. From day 11 to day 14, medium was changed into N2B27 medium supplemented with Sonic Hedgehog (SHH) (25 nmol/L, Prospec). Later, medium was changed into basic RPE medium consisting of DMEM/F-12 supplemented with 0.1 mmol/L non-essential amino acids, 1 mmol/L GlutaMAX, 1% N2 supplement and 5% Knockout Serum Replacement.

For Activin A treatment, the medium was changed to DMEM supplemented with 0.1 mmol/L non-essential amino acids, 1 mmol/L GlutaMAX, 20% Knockout Serum Replacement (Invitrogen), and Activin A (100 nmol/L, Prospec) from day 10 to day 20. Later medium was changed into the basic RPE medium as described above.

### Production of lentivirus and retrovirus

For generating lentiviral reporters, DNA fragments as described in Fig. 1A were obtained by PCR amplification from H1 hESC-derived genomic DNA template and cloned into pGreenZeo lentiviral vector (System bioscience). Corresponding packaging plasmids are pMDL, pCMV_VSVG and pRSV_REV. For transcription factors mediated lineage conversion, the human cDNAs listed in Fig. 3A were cloned into the pMX-gateway vector (Clontech). Coding region for mCherry was cloned as control. Corresponding packaging plasmids are pCMV-GAG-Pol and pCMV-VSV-G. HEK293T cells were seeded at a density between 6.0–8.5 × 10^4^ cells/cm^2^ and transfected by Lipofectamine 2000 (Invitrogen) 16 h later. Individual supernatants containing virus were harvested at 48 h and 72 h post-transfection and filtered with 0.45 μm PVDF membrane (Millipore).

### Conversion of human fibroblasts into Best1::GFP^+^ cells

Human foreskin fibroblasts (HFF-1, HFF-693) were plated on Matrigel-coated six-well plates at 75,000 cells per well. The next day, cells were infected with an equal ratio of a combination of eight retroviruses encoding PAX6, RAX, CRX, MITF-A, OTX2, NRL, KLF4 and c-MYC (8F) as well as pGZ-BEST1-GFP lentivirus. The plates were infected by spinfection of the cells at 1850 r/min for 1 h at room temperature in the presence of polybrene (4 μg/mL) and put back in the incubator without medium change. 24 h later, the medium was switched to CDF12 medium with medium changes every day. Cells were covered with 2% ESC qualified-Matrigel diluted in N2B27 medium at day 7. After overnight incubation, fresh RPE medium without Matrigel was added and changed every other day. At day 21, Best1::GFP^+^ colonies were picked and cultured in Matrigel coated 12 well plates, followed by 10 days treatment with 100 ng/mL Activin A or 500 nmol/L RA plus 25 ng/mL SHH in base RPE medium.

### Immunofluorescence staining

Cells were fixed using 4% paraformaldehyde in PBS at 4°C for 1 h. After fixation, cells were exposed to 0.3% Triton X-100 in PBS for 5 min at RT. Cells were blocked with 5% BSA in PBS for 30 min and incubated with primary antibody for 1 h at RT or overnight at 4°C. Washing was conducted with PBS followed by incubation with a corresponding secondary antibody for 1 h at RT. DAPI, was used to stain nuclei. Primary antibodies were obtained from the following sources: Mouse anti-Pax6 (1:1000, DSHB); Mouse anti-MITF (1:50, Millipore); Rabbit anti-ZO-1 (1:200, Sigma); Mouse anti-Best1 (1:200, Millipore).

### Quantitative PCR (qPCR)

Total cellular RNA was isolated using Trizol reagent (Invitrogen), or RNeasy Micro Kit (Qiagen) for a small amount of cell samples according to the manufacturer’s recommendations. Total RNA was treated with 2 μg of DNase 1 (Invitrogen) and used for cDNA synthesis using iScript reverse transcription supermix (Bio-Rad). Real-time PCR was performed using the SYBR Green PCR Master Mix (Applied Biosystems). The expression levels of respective genes were normalized to corresponding *GAPDH* values and are shown as fold change relative to the value of the control sample. All the assays were performed in triplicate. Primer sequences are listed in Table S1.

### Flow cytometry analysis and cell sorting

H9 hESCs were infected with lentiviral Best1::GFP reporter and then underwent RPE differentiation. Cells were harvested using TrypLE (Invitrogen), washed once with PBS and resuspended with 1× PBS/10% FCS medium. A minimum of 10,000 cells in the living population were analyzed by using a BD LSRII flow cytometry machine equipped with five different lasers and the BD FACSDiva software. Percentages of Best1::GFP^+^ cells are presented after the subtraction of isotype background and refer to the total living population analyzed. Results are representative of at least three independent experiments with a minimum of two technical replicates per experiment. For cell sorting, cells were treated as described above and sorted with a BDAria II FACS sorter (BD Biosystems).

## Electronic supplementary material

Below is the link to the electronic supplementary material.

Supplementary material 1 (PDF 8 kb)

## Abbreviations

AMD: age-related macular degeneration
ESC: embryonic stem cell
FACS: fluorescence activated cell sorting
iPSC: induced pluripotent stem cell
MEF: mouse embryonic fibroblast
RA: retinoic acid
RPE: retinal pigment epithelium
SHH: Sonic Hedgehog
TF: transcription factor
