# Supplementary material for: Direct conversion of human fibroblasts into retinal pigment epithelium-like cells by defined factors
Source: Protein Cell. 2014 Jan 29;5(1):48–58. doi: 10.1007/s13238-013-0011-2 (PMC3938849; doi:10.1007/s13238-013-0011-2)
Supplement: Supplementary file 1 — Supplementary material 1 (PDF 8 kb) [file 13238_2013_11_MOESM1_ESM.pdf]

Supplementary Table1. List of primers employed in this study

Gene	5'oligo	3'oligo
Best1	CTGGGCTTCTACGTGACGC	AGGTTCTCGTACTGGTTCCAC
cMyc	CGGGCGGGCACTTTG	GGAGAGTCGCGTCCTTGCT
CRALBP	AAGCTGGCTACCCTGGTGT	TGAAGCAATATGCCTGCAAGA
Crx	GCCCCACTATTCTGTCAACG	GTCTGGGTACTGGGTCTTGG
GAPDH	GGACTCATGACCACAGTCCATGCC	TCAGGGATGACCTTGCCCACAG
Klf4	AGCCTAATTGATGGTGCTTGGT	TTGAAAACTTTGGCTTCCTTGTT
Lhx2	ATGCTGTTCCACAGTCTGTCG	GCATGGTCGTCTCGGTGTC
Mitf	TGGTTTTCCCACGAGCTATTTT	GCACAGAGTCAATTTCCTGGT
Nrl	GGCTCCACACCTTACAGCTC	GGCCCATCAACAGGGACTG
Otx2	CAAAGTGAGACCTGCCAAAAAGA	TGGACAAGGGATCTGACAGTG
Pax6	AGTGAATCAGCTCGGTGGTGTCTT	TGCAGAATTCGGGAAATGTCGCAC
Pedf	TTCAAAGTCCCCGTGAACAAG	GAGAGCCCGGTGAATGATGG
Rax	GAATCTCGAAATCTCAGCCC	CTTCACTAATTTGCTCAGGAC
Rpe65	CCTGCTGGTGGTTACAAGAAA	CCTGCCTGTTACATGAGCTGT
Six3	CAAGGAGTCTCACGGCAAG	GCAATGCGTCTTCTGCTCG
Six6	GCCCTCAACAAGAATGAGTCG	GCCTCCTGGTAGTGTGCTTC
Tyr	TGCACAGAGAGACGACTCTTG	GAGCTGATGGTATGCTTTGCTAA
Tyrp2	AACTGCGAGCGGAAGAAACC	CGTAGTCGGGGTGTACTCTCT
